# Using web-based videos to improve inhalation technique in COPD patients requiring hospitalization: A randomized controlled trial

**DOI:** 10.1371/journal.pone.0201188

**Published:** 2018-10-16

**Authors:** Wolfram Windisch, Sarah Bettina Schwarz, Friederike Sophie Magnet, Michael Dreher, Claudia Schmoor, Jan Hendrik Storre, Verena Knipel

**Affiliations:** 1 Department of Pneumology, Kliniken der Stadt Köln gGmbH, Universität Witten/Herdecke, Cologne, Germany; 2 Department of Pneumology, University Hospital RWTH Aachen, Aachen, Germany; 3 Clinical Trials Department, Faculty of Medicine and Medical Center—University of Freiburg, Freiburg, Germany; 4 Department of Intensive Care, Sleep Medicine and Mechanical Ventilation, Asklepios Fachkliniken Munich-Gauting, Munich-Gauting, Germany; 5 Department of Pneumology, University Medical Hospital, Freiburg, Germany; Public Library of Science, UNITED KINGDOM

## Abstract

**Background:**

Inhalation errors frequently occur in patients receiving inhalation treatment, which can significantly impair treatment success. While this underscores the importance of inhalation training, the role of modern web-based instructional videos has not yet been investigated.

**Methods:**

A randomized controlled trial using standardized checklists (10 items: preparation, N = 3, inhalation routine, N = 6, and closure of inhalation, N = 1) was carried out to determine the relative effects of web-based, device-specific videos versus standard personal instruction on reducing multiple (≥2) inhalation errors in severe COPD patients requiring hospitalisation. Investigators assessing inhalation errors were blinded to the intervention.

**Results:**

Multiple handling errors were recorded at baseline in 152 out of 159 patients (95.6%). Each teaching method led to a similar reduction in errors (videos: from 4.2±1.6 to 1.5±1.5 errors; personal instruction: from 3.8±1.5 to 1.3±1.6; p<0.0001), although non-inferiority of web-based video teaching could not be confirmed statistically due to an unpredictably high number of patients in both groups still making multiple handling errors (44.0% versus 40.3%, mean difference 3.7%; 95%CI [-12.0–19.4%]).

**Conclusion:**

Multiple inhalation errors regularly occur in severe COPD patients requiring hospitalisation. Web-based video teaching is capable of reducing inhalation errors. However, compared to personal instruction non-inferiority could not be established. This was due to an unexpectedly high number of patients with persisting inhalation errors despite training.

**Trial registration:**

Clinical trial Registration: German Clinical Trial Register, DRKS 00004320.

## Background

Chronic obstructive pulmonary disease (COPD) ranks among the most widespread of chronic diseases contributing to morbidity and mortality across the world.[1,2] Inhalation therapy with drugs delivered in aerosol form directly to the lungs has become the method of choice for long-term treatment, since this allows high drug concentrations at the target site, with negligible or acceptable systemic side-effects.[3–7] Bronchodilators such as beta-2-adrenergic agonist and anticholinergics, with the addition of inhaled corticosteroids (ICS) or not, are currently recommended for inhalative treatment in COPD patients.[1] Furthermore, the more rapid onset of action in inhalation therapy serves as an advantage over systemic treatment.[3,4,7]

An optimal inhalation technique is essential to the inhaled drug being successfully deposited in the lungs.[3,7–15] While proper deposition is partly dependent on the medication itself (e.g. type of inhaler device, particle size), it also relies on the patient performing a truly correct inhalation manoeuvre.[3,8,9] Thus, both inhalation technique and performance form integral parts of drug deposition within the lungs.[3,6–10,15–17]

It is widely accepted that optimal inhalation therapy can be hindered by poor patient adherence to treatment, whether it be intentional (patient’s beliefs, doubts, fears of adverse effects) or non-intentional (when the patient forgets to use the inhaler device, or has no access to it).[6,18] Importantly, even if the patient is willing and able to use the inhaler, a number of studies have identified that patient-related errors in inhalation techniques, particularly in those with COPD, are common and associated with reduced disease control.[10–13,19] Therefore, inhalation technique training forms an essential basis for the optimal treatment of patients with COPD. Thereby, in-person instructions have been clearly shown to be useful in patients with obstructive airway diseases following exacerbation and hospitalization.[20] Since 2013, the German Airway League (Deutsche Atemwegsliga) has provided web-based, device-specific videos demonstrating proper inhalation techniques.[14,21–24] These videos aim to facilitate the teaching of correct inhalation and carry the following advantages: (i) no instructor is needed; (ii) they are universally accessible via the internet; (iii) they can be watched repeatedly; (iv) they are available free of charge if used for non-profit reasons. Based on these favourable aspects, the teaching videos presumably serve as a practical, easy-to-use, time-saving resource. Therefore, the present study aimed to investigate the role of web-based videos in teaching the correct inhalation technique to patients with COPD.

## Methods

This prospective single-centre randomized controlled trial was conducted at the Department of Pneumology, Cologne Merheim Hospital, University of Witten/Herdecke, Germany. The study was approved by the ethics committee of the University of Witten/Herdecke and performed in accordance with the Declaration of Helsinki. The study was registered at the German Clinical Trials Register (Registration number: DRKS 00004320; Date of registration: 12.09.2012; Date of first enrollment: 05.12.2012). Informed written consent was obtained from all subjects.

### Patients

COPD patients were enrolled between December 2012 and June 2014 and were screened for eligibility during a hospital stay for recovery from an exacerbation and were included if they did not meet any of the following exclusion criteria: acute respiratory failure as defined by a pH <7.35, breathing frequency >23/min at rest, need for supplemental oxygen of >3L/min. Neurologic, orthopaedic or cognitive conditions hindering inhalative treatment also served as exclusion criteria. Only patients with an established GOLD-criteria based diagnosis of COPD were included if they had used long-term inhalative treatment prior to hospital submission. Patients who used either a pressurized metered-dose inhaler (pMDI), dry powder inhaler and/or soft mist inhaler were included.

### Study design

Full bodyplethysmography (ZAN 500; nSpire, Germany) [25–29] was performed to confirm diagnosis of COPD and assess the severity of airway obstruction. Diurnal arterial blood samples were taken from the arterialized earlobe during individually-tailored delivery of oxygen (ALB800 Flex, Radiometer, Denmark).

Individual subject-related errors were assessed using standardized check-lists (see below). Participants with 2 or more errors were deemed to perform the inhalation process incorrectly without doubt (multiple handling errors). All of these participants, therefore, received inhalation training. For this purpose, subjects were randomized amongst two groups, one in which they had to undergo a personal instruction in the full attendance of a physician, and the other in which they had to undergo web-based video teaching.[30]

The personal instruction was standardized and performed by one physician in full attendance. First, subjects were verbally instructed how to use the inhaler and were, subsequently, shown the correct inhalation process using a demo device. Next, participants were requested to practice all the steps of correct inhalation with teach-back. Here, particular emphasis was placed on the steps that the subjects had originally performed incorrectly. Finally, all participants had the opportunity to ask any outstanding questions.

For the web-based video training, subjects were shown web-based videos [21–23] on a tablet device (iPad^®^, Apple Inc., Cupertino, USA). For this purpose, subjects were allowed to watch the video as many times as they deemed necessary. Instructions on how to get the videos started were provided by a physician who did not interact any further with the subject during the inhalation training process or respond to final questions.

After 24 hours, individual patient-related errors were assessed again using the same standardized check-list. In order to avoid investigator bias, both the initial and post-teaching error assessments were performed by the same investigator. Investigators performing the assessments were blinded to the type of teaching intervention. Thus, there were two different investigators involved for each patient: one for the assessment of patient-related inhalation errors (before and after teaching) and one for the teaching intervention. These investigator roles were exchanged in a random order.

### Web-based teaching videos provided by the German Airway League

The teaching videos (1:42 to 3:11 min:sec) are provided by the German Airway League (www.atemwegsliga.de).[14] They can be downloaded free of charge if used for non-profit purposes. Regularly updates video screens are available for all currently available inhaler devices. The videos combine a device-specific illustration of correct inhalation, spoken text passages, and visual information with persons demonstrating the correct inhalation process. The original language is German, but the videos have also been translated into Arabic, English, Russian, Slovakian, and Turkish with further translations being in progress.

### Checklists

Checklists for the assessment of correct inhalation were recently developed by the German Airway League [7] and can be downloaded free of charge (www.atemwegsliga.de). The checklists were standardized to allow comparability amongst patients. Three major steps of the inhalation process were covered by each checklist: 1. inhalation preparation (3 items); 2. inhalation routine (6 items); and, 3. closure of inhalation (1 item). The checklists were originally developed in German, but have also been professionally translated into English.[7]

### Statistical analysis

The purpose of the study was to show that web-based video teaching is not inferior to personal instruction with respect to the probability of a severe handling error persisting, as defined by ≥2 errors described on the checklists. For the purpose of sample size calculation it was assumed that following personal instruction, the probability of a severe handling error persisting would be 0.05. Non-inferiority was assigned if the probability that a severe handling error persisted after web-based video teaching was not higher than 0.15. Thus, the limit for non-inferiority was 0.1. At least 75 patients per group were needed in order to show non-inferiority of the two methods on a one-sided alpha level of 0.025 with a power of 80%.[30] Non-inferiority was statistically shown when the upper limit of the two-sided 95% confidence interval (CI) of the difference between error probabilities after subtracting video teaching from personal instruction was lower than 0.1. If the upper limit was lower than 0, superiority of the web based video teaching was shown. The CI was calculated using the normal approximation of the binomial distribution.

Baseline characteristics were descriptively compared between teaching methods. The results of the checklists are descriptively presented for each device. Teaching results and baseline measurement of handling errors were compared by calculating the mean difference with 95% CI and performing a paired t-test separately for personal instruction and web-based video teaching. As an additional post-hoc analysis, the number of handling errors was compared after video teaching versus personal instruction using a linear regression model including besides randomized instruction method the number of handling errors at baseline for adjustment.

## Results

**Fig 1:**
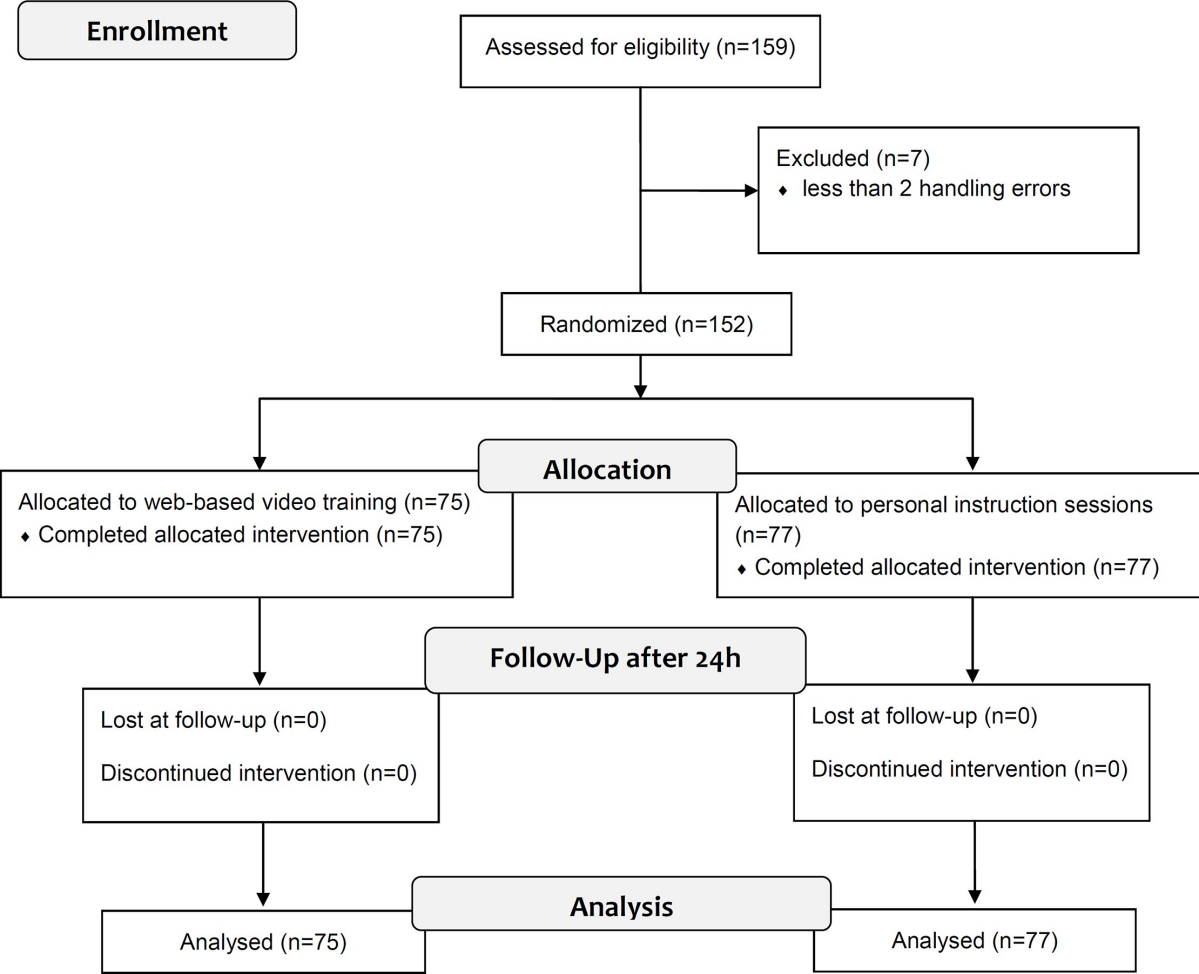
Flowchart of the study design according to the CONSORT 2010 Statement [31].

A total of 159 consecutively-recruited patients were screened. Only seven patients did not have multiple inhalation errors (4.4%), leaving 152 patients with multiple handling errors (95.6%) to be subsequently randomized to the two different teaching groups (Fig 1 and Table 1).

**Table 1:** Demographic data and GOLD stages (N = 152).

Characteristics	Web-based video N = 75	Personal instruction N = 77
**Age** (years), mean ± SD	66.6±7.7	68.2±9.1
**Male** (% of total)	45.3	52.0
**BMI** (Kg/m^2^), mean ± SD	26.9±7.1	26±6.9
**Smoking** (pack years), mean ± SD	51.9±36.2	47.6±27.6
**High School** (%)	8.0	10.4
**Secondary School** (%)	18.7	22.1
**Primary School** (%)	66.7	62.3
**Special-needs School** (%)	2.7	0
**No School** (%)	4.0	2.6
**Exacerbation history** (per year), mean ± SD	2.6±1.3	2.6±1.7
**CAT Score**	25.2±6.9	24.9±6.8
**GOLD Stage 1** (%)	2.7	0
**GOLD Stage 2** (%)	21.3	20.8
**GOLD Stage 3** (%)	37.3	40.3
**GOLD Stage 4** (%)	38.7	39.0
**GOLD Stage A** (%)	0	0
**GOLD Stage B** (%)	2.7	5.2
**GOLD Stage C** (%)	1.3	0
**GOLD Stage D** (%)	96.0	94.8
**FEV**_**1**_ (% pred), mean ± SD	39.2±17.4	37.0±16.4
**FEV**_**1**_**/FVC** (%),mean ± SD	49.1±13.1	47.2±11.8
**FVC** (% pred), mean ± SD	63.9±22.2	62.6±19.6
**RV** (% pred), mean ± SD	187.6±72.8	182.6±81.6
**TLC** (% pred), mean ± SD	110.5±22.7	107.2±28.3

Prior to the study, the source of instruction for handling the inhalation device for both teaching methods (web-based video teaching/personal instruction) was as follows: outpatient physician (44/40), medical staff (16/14), no instructions (6/7), inpatient physician (6/5), written instructions for use (3/7), pharmacist (0/2) and family members (0/2). This information revealed that 25 patients were not instructed by medical specialists (16.4%). The mean number per patient of baseline, device-specific inhalation errors in patients with severe handling errors are presented in Table 2.

**Table 2:** Device-specific inhalation errors at baseline (N = 152).

		Mean number of errors per patient		
	Patients N	Preparation (out of 3 steps)	Inhalation (out of 6 steps)	Closure (out of 1 step)
**pMDI**	47	0.53	3.21	0
**Handihaler**	43	0.67	2.49	0.77
**Turbohaler**	24	1.04	2.92	0.25
**Breezhaler**	10	0.80	2.80	0.60
**Diskus (Accuhaler)**	9	1.11	3.22	0.67
**Respimat**	8	1.25	2.50	0.13
**Aerolizer**	7	0.57	2.71	0.57
**Novolizer**	4	0.75	3.50	1.00

pMDI = pressurized metered dose inhaler

**Fig 2:**
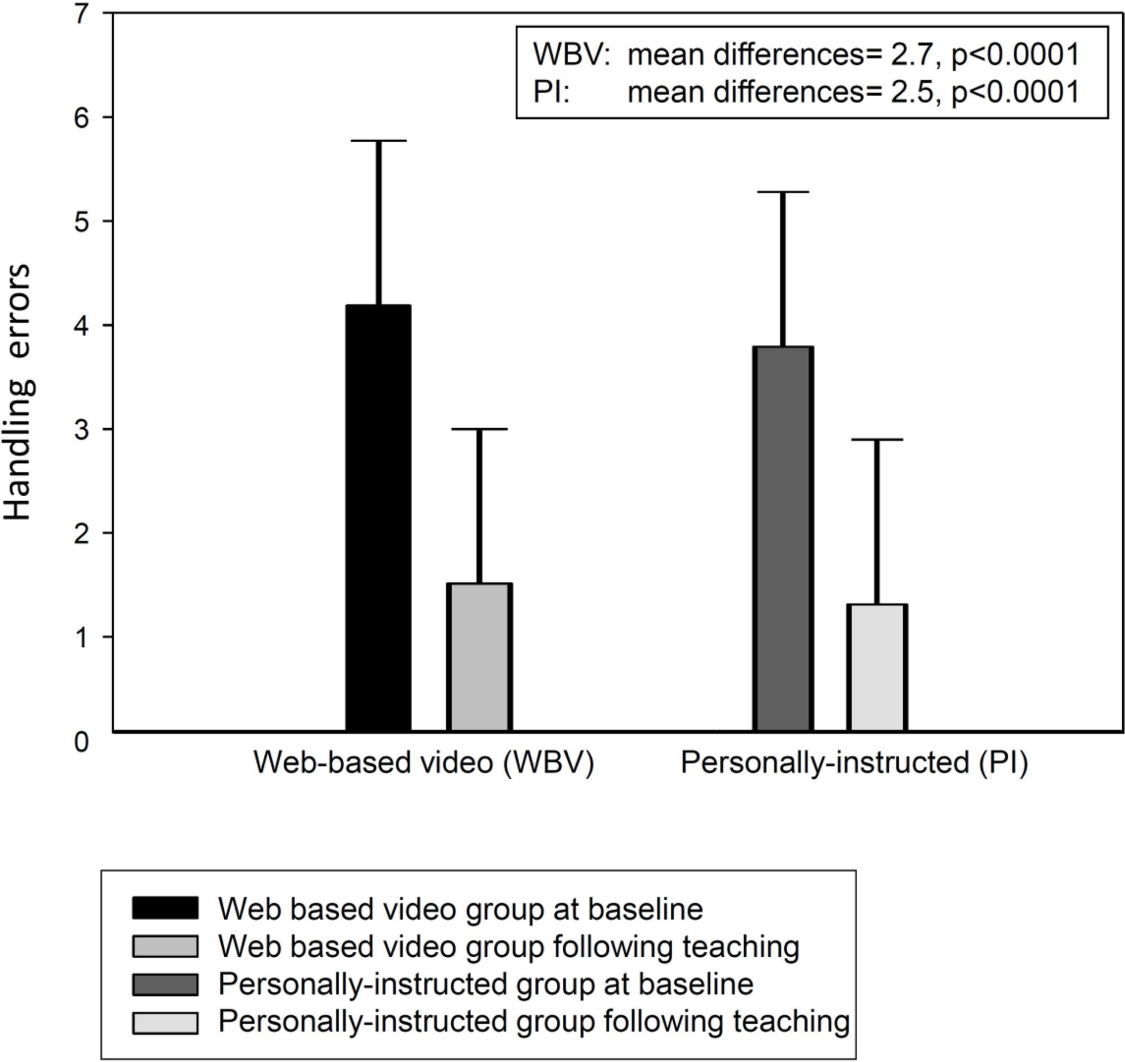
Inhalation error rate at baseline and following teaching (N = 152).

Both interventions were associated with a significant reduction in inhalation errors. The mean difference was 2.5 (95%-CI [2.2, 2.9], p<0.0001) for personal instruction and 2.7 (95%-CI [2.3, 3.1], p<0.0001) for web-based video teaching (Fig 2). However, 42.1% (N = 64) of all patients with multiple handling errors at baseline still made multiple handling errors following teaching. In this regard, the probability of multiple handling errors was not different between the web-based video teaching and personal instruction groups (0.440 versus 0.403, respectively; mean difference 0.037; 95%CI [-0.120–0.194]). The upper limit of the CI was 0.194 and, therefore, higher than 0.1. For this reason, non-inferiority of web-based video teaching to personal instruction could not be formally established, even though both teaching methods showed similar results. This was possibly due the fact that the proportion of patients with multiple post-teaching handling errors was much higher than what was estimated during study planning and subsequently used for sample size calculation. In the present study, the multiple handling error rate following teaching intervention was 0.421; however, for non-inferiority to have been established, the severe handling error would have had to be defined by at least 5 errors (Table 3), since this error rate occurred with the assumed probability of 0.05.

The mean difference in the number of handling errors after video teaching versus personal instruction adjusted for the number of handling errors at baseline was -0.07 (95%-CI [-0.52, 0.39], p = 0.76).

**Table 3:** 95% Confidence Intervals for the comparison of face-to-face consent discussion with web-based video teaching, with respect to the number of inhalation errors.

Number of errors	Web-based video teaching	Personal instruction	Difference	95% CI Lower limit	95% CI Lower limit
≥ 2	0.440	0.403	0.037	-0.120	0.194
≥ 3	0.213	0.143	0.070	-0.051	0.192
≥ 4	0.093	0.065	0.028	-0.057	0.114
≥ 5	0.053	0.052	0.001	-0.070	0.072

## Discussion

This is the first study to perform a randomized, controlled evaluation of the relative capacities of web-based video teaching versus personal instruction in improving inhalation technique in severe COPD patients with severe handling errors. The main results are as follows: Firstly, more than 95% of COPD patients, predominantly with advanced disease state and with frequent exacerbations, had multiple handling errors; secondly, both the web-based video teaching and personal instruction were each capable of significantly reducing the number of inhalation errors; thirdly, severe handling errors at baseline still persisted in 42% of patients following teaching; fourthly, there was no difference between web-based video teaching and personal instruction regarding the number of patients with multiple handling errors following teaching. However, non-inferiority for web-based video teaching could not be statistically established.

A recent real-life study reported that regardless of the devices used, handling errors occurred in over 50% of COPD patients, where most suffered from a moderate form of the disease.[32] Handling errors in this study were associated with an increased rate of severe COPD exacerbation. Based on this data it was concluded that training patients to use their inhaler correctly forms an integral part of COPD management.

The present study builds on these findings by showing that >95% of patients with predominantly severe COPD and exacerbation requiring hospitalisation had multiple handling errors. Inhalation errors were also distributed differently across different inhalers depending on preparation, performance, and closure of the inhalation process. However, the present study was not aimed to compare different devices, but the current device-specific error list may help in the design of future studies investigating device-specific inhalation errors.

Despite training, more than 40% of patients in the current study with multiple inhalation errors were still unable to use the inhalation devices correctly. In 69% of the study patients, the highest level of education was primary school or less. However, it remains unclear if cognitive and/or physical dysfunction were responsible for patients remaining unable to correctly inhale, despite teaching. Nevertheless, the present study emphasizes that despite teaching, some patients with the severe forms of COPD simply cannot inhale correctly, and that this is likely to underlie the subsequent exacerbation episodes requiring hospitalisation. Whether a special, extended training program during an earlier stage of the disease could lead to an improvement in inhalation handling remains to be elucidated. In this respect, it should be noted that a recent study emphasised the need to repeat training instructions at least three times in order to achieve effective inhalation skills in both asthma and COPD patients.[33]

The present study clearly shows the potential of web-based video teaching in improving inhalation handling. Thereby, web-based video teaching was fairly comparable to personal instruction in terms of effectively improving inhalation handling. Therefore, web-based video teaching serves as a useful option if medical specialists are not available, or do not have time to provide patients with instructions. Furthermore, a major advantage of web-based video teaching is that it is internet based and therefore not dependent on time or location. Further advantages of the videos are that they are free of charge, cover all available devices with regular updates, are short and practical, and are available in different languages. In addition, a recent study demonstrated the efficacy of the virtual Teach-to-Goal™ adaptive learning module, which is used to teach the MDI inhaler technique to patients with asthma and COPD.[34] This approach differs from the current videos as it is more complex and, thereby, incorporates feed-back. In line with the present study, however, this approach also provides the advantages of electronic availability.

There are, however, some limitations of the study that need to be addressed. Firstly, during the final analysis the sample size turned out to be too low to establish non-inferiority of the web-based video teaching. This was probably due to an unexpectedly high number of patients with persisting severe inhalation errors, despite teaching. Non-inferiority could only have been established if the severe handling error was defined by at least 5 out of 10 checklist errors, which would have led to an error probability of 0.05 for the patients, as assumed in study planning. Therefore, it is not possible to conclude that web-based video teaching can completely replace personal instruction. Nevertheless, the purpose of these videos is not to replace personal instruction, but rather to complement the current teaching practices. To this end, it was recently shown that teaching correct inhalation techniques is essential in obstructive airway diseases [35]. Nevertheless, both techniques used in the current study turned out to be effective in improving inhalation device handling. Secondly, the duration of teaching was not assessed prospectively. Therefore, it remains unclear as to whether web-based video teaching has a time-saving element. However, since web-based video teaching in the present study was not accompanied by a respiratory specialist, it saves time for professionals. Thirdly, the time span between the initial evaluation of the inhalation process and its second evaluation following teaching was 24 hours and therefore rather short. As the video screen would be available permanently, but personal instruction would not, using video screens for inhalation training could be advantageous when considering successful long-term inhalative treatment. This should be investigated in the future. Fourthly, the results of this study may only be valid for advanced COPD patients who require hospitalisation, even though web-based video teaching seems likely to be successful in less-severe COPD patients. Finally, a standardized teach-to goal approach was not used for the purpose of the study, and this was true for both teaching techniques. This might also explain the persistently high failure rates following teaching. Therefore, future studies should also address if failure rates of inhalation could be further improved by addressing each step of inhalation using a forth and back approach to goal. This appears to be particularly important in view of the observation that feed-back is suggested to be highly important in achieving best inhalation performance [34].

In conclusion, severe inhalation errors regularly occur in COPD patients who have exacerbations that require hospitalisation. Inhalation training significantly improves inhalation handling in these patients. However, despite training, more than 40% of these patients are not able to correctly use devices for inhalative treatment. Both, web-based video teaching and personal instructions when used for inhalation training were capable of significantly improving the inhalation technique. However, non-inferiority of web-based video teaching could not be statistically confirmed due to the unexpectedly high number of patients remaining unable to correctly inhale post-training in both interventions. Therefore, further studies are needed to verify the usefulness of web-based video inhalation teaching.

## Conclusion

In patients with severe COPD requiring frequent hospitalization this trial indicates that most patients present with severe handling errors of inhalative treatment. Furthermore, more than 40% of these patients are still not able to correctly inhale despite inhalation training. Officially provided web-based videos are nearly as effective as standard personal instruction accompanied by a physician in improving the inhalation technique in these patients.

## Supporting information

S1 File Study design original. (DOC)

S2 File Consort 2010 checklist. (DOC)

S3 File Study design Engl. Translated Study design.
(DOC)
