# Supplementary material for: Using web-based videos to improve inhalation technique in COPD patients requiring hospitalization: A randomized controlled trial
Source: PLoS One. 2018 Oct 16;13(10):e0201188. doi: 10.1371/journal.pone.0201188 (PMC6191087; doi:10.1371/journal.pone.0201188)
Supplement: S1 File — (DOC) [file pone.0201188.s001.doc]

**Studiendesign - Version 16. April 2012**

**Vermeidung von Handhabungsfehlern bei der inhalativen Therapie:**

**Ärztliche Einweisung versus Aufklärungsfilm**

**Eine randomisierte, verblindete Studie**

***Wolfram Windisch^1^, Sarah Bettina Schwarz^1^, Friederike Sophie Magnet^1^, Michael Dreher^2^, Claudia Schmoor^3^, Jan Hendrik Storre^4,5^, Verena Knipel^1^***

^1^Kliniken der Stadt Köln gGmbH – Lungenklinik, Universität Witten/Herdecke, Ostmerheimer Straße 200, 51109 Köln

^2^Division of Pneumology, University Hospital RWTH Aachen, Germany

^3^Clinical Trials Unit, Faculty of Medicine and Medical Center - University of Freiburg, Germany.

^4^Department of Intensive Care, Sleep Medicine and Mechanical Ventilation, Asklepios Fachkliniken Munich-Gauting, Germany

^5^Department of Pneumology, University Medical Hospital, Freiburg, Germany

Lungenklinik Merheim, Kliniken der Stadt Köln gGmbH, Köln, Germany

####
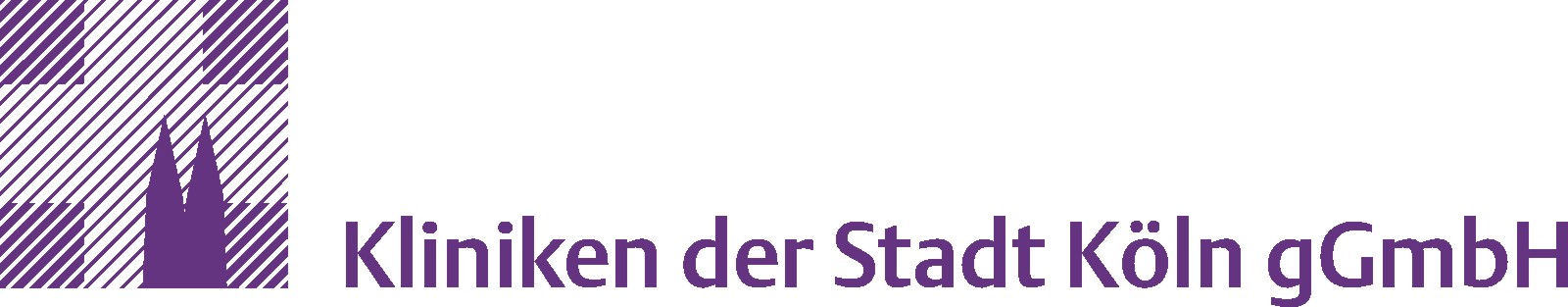


Dr. med. Jan Hendrik Storre

Lungenklinik Merheim

Kliniken der Stadt Köln gGmbH

Ostmerheimer Str. 200

D – 51109 Köln

Tel.: +49 221 8907-0; Fax.: +49 221 8907 3048

e-mail: [storrej@kliniken-koeln.de](mailto:storrej@kliniken-koeln.de)

### Hintergrund

Die Verwendung von Inhalatoren stellt bei unterschiedlichen Atemwegserkrankungen eine entscheidende Säule der Therapie dar. Die Handhabung der verschiedenen Inhalatoren ist jedoch komplex.^1^ Trotz Training der Inhalationstechnik fiel bei älteren Patienten mit fortgeschrittener COPD eine hohe Fehlerquote in der Anwendung der Therapie auf.^2^

In einer aktuellen Untersuchung von Melani und Mitarbeitern konnte nachgewiesen werden, dass Handhabungsfehler im Alltag auch in spezialisierten Zentren weit verbreitet sind.^3^ Die Studie untersuchte Patienten, die an einer COPD oder Asthma litten und an einem pneumologischen Zentrum ambulant angebunden waren. Eine richtig durchgeführte Inhalationstechnik ist jedoch die Voraussetzung für einen Therapieerfolg.^1^ Die häufigsten Inhalationsfehler traten in der aktuellen Untersuchung bei Menschen mit höherem Lebensalter und geringerer Schulbildung auf, sowie aufgrund der fehlenden Einweisung durch Ärzte oder medizinischer Fachpflege. Handhabungsfehler führten zu einem höheren Risiko für Krankenhausaufenthalte, Einweisungen in die Notaufnahme, systemischer Behandlung mit Kortikosteroiden und antimikrobiellen Therapien sowie einer schlechteren Krankheitskontrolle.^3^

Somit zeigen diese Ergebnisse eindrücklich, dass Patienten trotz Anbindung an ein fachärztliches Zentrum Defizite in der Anwendung der inhalativen Medikamente zeigen, welche zur Optimierung der Therapie verbessert werden muss. Die Deutsche Atemwegsliga e.V. hat aktuell auf Ihrer Homepage Videomaterial über die richtige Inhalationstechnik verschiedener Medikamente veröffentlicht, welche eine stets abrufbare Informationsquelle für Patienten und medizinisches Personal darstellt.

### Ziel des Projekts

In der aktuellen Studie von Melani und Mitarbeitern zeigte sich, dass eine fehlende Einweisung in die Inhalationstechnik durch Ärzte oder medizinische Fachkräfte mit einem erhöhten Risiko für Handhabungsfehler bei Inhalatoren im Alltag assoziiert war.^3^

Das Ziel des aktuellen Projektes ist es zu prüfen, ob ein Aufklärungsfilm [Deutsche Atemwegsliga e.V., <http://www.atemwegsliga.de/richtig-inhalieren.html>] einem qualifizierten ärztlichen Einweisungsgespräch hinsichtlich des Auftretens erheblicher Handhabungsfehler nicht unterlegen oder sogar überlegen ist und somit gegebenenfalls das Aufklärungsgespräch ersetzen kann.

**Patienten**

Eingeschlossen werden COPD Patienten, die bereits mit einem Dosieraerosol (engl. MDI= metered dose inhaler) oder einem Trockenpulver-Inhalator (engl. DPI= dry powder inhaler) therapiert werden und stationär in die Lungenklinik Merheim, Kliniken der Stadt Köln, aufgenommen werden.

Die Patienten werden über die Studie von einem Arzt aufgeklärt und haben vor Studieneinschluss ihr schriftliches Einverständnis zur Teilnahme an der Studie gegeben.

Ausgeschlossen werden Patienten, die aufgrund kognitiver, neurologischer oder orthopädischer Erkrankungen an einer korrekten Durchführung einer eigenständigen Inhalation gehindert sind. Ebenso die Patienten, die eine nahezu richtige Technik anwenden (Fehlerindex < 2 Punkte).

##### Studiendesign

Nach Aufklärung der Patienten und Studieneinschluss werden die Patienteninformationen (Anhang) zur Erhebung der demographischen und anamnestischen Daten aufgenommen. Es erfolgt ebenso die Durchführung einer Ganzkörperplethysmographie. Im Anschluss erfolgt eine Analyse über die richtige Handhabung der verwendeten Inhalationsgeräte anhand der Checklisten (Anhang). Sollten die Patienten mehr als einen MDI oder DPI verwenden, so erfolgt die Beurteilung beider oder mehrerer Medikamente anhand der verschiedenen Fragebögen. Es werden sowohl Dauer- als auch Bedarfsmedikamente berücksichtigt.

Im Anschluss erfolgt eine Randomisierung, bei der die Patienten entweder durch ein ärztliches Einweisungsgespräch oder einen Aufklärungsfilm [Deutsche Atemwegsliga e.V., http://www.atemwegsliga.de/richtig-inhalieren.html] in der richtigen Inhalationstechnik geschult werden. Dies erfolgt jedoch nur für ein Inhalationsgerät, und zwar das Gerät mit der höchsten Fehlerquote. Sollte bei der Verwendung von mehreren Inhalationsgeräte eine gleiche Anzahl von Fehlern vorliegen, so entscheidet der Patient, bei welchem Gerät im rahmen der wissenschaftlichen Untersuchung die Einweisung durch den Arzt oder Film erfolgen soll.

Am Folgetag wird 24 Stunden nach der Schulung eine Re-Evaluation des Lernerfolgs durch einen hinsichtlich der Intervention (Aufklärungsfilm versus ärztliche Einweisung) verblindeten Arzt mit einer erneuten Analyse über die richtige Handhabung der Inhalationsgeräte anhand der Checklisten durchgeführt. Um eine unterschiedliche Beurteilung auszuschließen, wird derselbe eingesetzt, der auch die initiale Handhabung der Inhalation am Vortag evaluierte.

##### Fragebogen und Checklisten

In Analogie zur Studie von Melani et al. wurden Checklisten zur richtigen Durchführung der Inhalationstechnik entwickelt.^3^ Für die verschiedenen MDI und DPI wurde aufgrund ihrer unterschiedlichen Handhabung pro Gerätetyp eine Checkliste erstellt. Um eine vergleichbare Evaluation der verschiedenen Inhalationsgeräte zu ermöglichen, wurden für jedes Gerät zehn Fragen entwickelt, wobei sich jeweils drei Fragen auf die richtige Vorbereitung, sechs Fragen auf die richtige Anwendung und eine auf den richtigen Abschluss der Inhalationstechnik beziehen (Anhang). Aufgrund dieser Aufteilung ist eine Subanalyse der Fehlerquellen möglich.

Die Patienten werden in die Studie eingeschlossen ab einer Summe von zwei Fehlerpunkten, was als erheblicher Handhabungsfehler definiert wird.

##### Statistische Beratung

Das primäre Zielkriterium der Studie zum Vergleich der beiden Aufklärungsmethoden ist das Vorliegen eines erheblichen Handhabungsfehlers, definiert als ≥2 Fehlerpunkte bei dem untersuchten Gerät.

##### Die Fallzahlkalkulation wird auf Basis des primären Zielkriteriums durchgeführt. Es soll gezeigt werden, dass ein Aufklärungsfilm nicht schlechter ist als ein ärztliches Einweisungsgespräch. Es wird angenommen, dass nach einem ärztlichen Einweisungsgespräch die Wahrscheinlich­keit pA für das Vorliegen eines erheblichen Handhabungsfehlers bei 0.05 liegt. Der Aufklärungsfilm wird als nicht schlechter als das Einweisungsgespräch angesehen, wenn danach die Wahrscheinlich­keit pF für das Vorliegen eines erheblichen Handhabungsfehlers bei maximal 0.15 liegt, d.h. die Nicht-Unterlegenheitsschranke wird auf 0.1 festgelegt. Um dies zum einseitigen Niveau α=2.5% mit Power von 80% unter der Annahme der Gleichheit der Methoden nachweisen zu können, werden 75 Patienten pro Gruppe, also insgesamt 150 Patienten, benötigt.

##### Für den Test der Hypothese pF – pA ≥ 0.1 gegen die Alternative pF – pA < 0.1 wird das zweiseitige 95% Konfidenzintervall für pF – pA berechnet. Wenn die obere Grenze dieses Intervalls kleiner als 0.1 ist, wird die Hypothese abgelehnt und auf Nicht-Unterlegenheit des Aufklärungsfilms geschlossen. Wenn die obere Grenze des Konfidenzintervalls kleiner als 0 ist, wird auf Überlegenheit des Aufklärungsfilms geschlossen.^4^ Die detaillierten Ergebnisse der Checkliste werden deskriptiv analysiert.

**Literatur**

1 Laube BL, Janssens HM, de Jongh FHC, et al. What the pulmonary specialist should know about the new inhalation therapies. Eur Respir J 2011; 37:1308-31.

2 Wieshammer S, Dreyhaupt J. Dry powder inhalers: which factors determine the frequency of handling errors? Respiration 2008; 75:18-25.

3 Melani AS, Bonavia M, Cilenti V, et al. Inhaler mishandling remains common in real life and is associated with reduced disease control. Respir Med 2011; 105:930-8.

4 Blackwelder WC. 'Proving the null hypothesis' in clinical trials. Control Clin Trials 1982; 3:345-53.
