# Supplementary material for: Using web-based videos to improve inhalation technique in COPD patients requiring hospitalization: A randomized controlled trial
Source: PLoS One. 2018 Oct 16;13(10):e0201188. doi: 10.1371/journal.pone.0201188 (PMC6191087; doi:10.1371/journal.pone.0201188)
Supplement: S3 File — Translated Study design. (DOC) [file pone.0201188.s003.doc]

**Study design - Version 16. April 2012**

**Using web-based videos to improve inhalation technique in COPD patients requiring hospitalization**

**A randomized controlled trial**

***Wolfram Windisch^1^, Sarah Bettina Schwarz^1^, Friederike Sophie Magnet^1^, Michael Dreher^2^, Claudia Schmoor^3^, Jan Hendrik Storre^4,5^, Verena Knipel^1^***

^1^Kliniken der Stadt Köln gGmbH – Lungenklinik, Universität Witten/Herdecke, Ostmerheimer Straße 200, 51109 Köln

^2^Division of Pneumology, University Hospital RWTH Aachen, Germany

^3^Clinical Trials Unit, Faculty of Medicine and Medical Center - University of Freiburg, Germany.

^4^Department of Intensive Care, Sleep Medicine and Mechanical Ventilation, Asklepios Fachkliniken Munich-Gauting, Germany

^5^Department of Pneumology, University Medical Hospital, Freiburg, Germany

Department of Pneumology, Kliniken der Stadt Köln gGmbH, Cologne, Germany

####
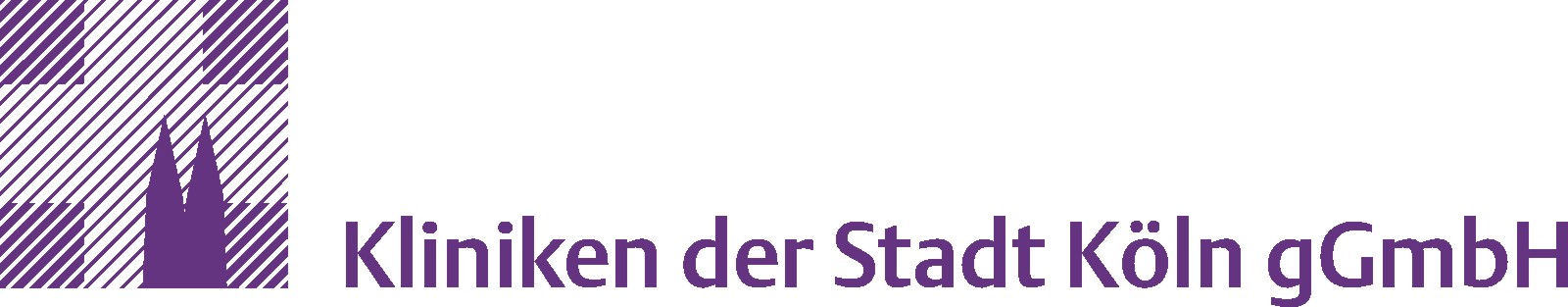


Dr. med. Jan Hendrik Storre

Lungenklinik Merheim

Kliniken der Stadt Köln gGmbH

Ostmerheimer Str. 200

D – 51109 Cologne

Tel.: +49 221 8907-0; Fax.: +49 221 8907 3048

e-mail: [storrej@kliniken-koeln.de](mailto:storrej@kliniken-koeln.de)

**Background**

Inhalation therapy with drugs delivered in aerosol form directly to the lungs has become the method of choice for long-term treatment. However, inhalation therapy is complex due to different devices.[1] Despite training of inhalation technique many patient-related errors in inhalation techniques, particularly in those with COPD, are common and associated with reduced disease control.[2] In a recent trial Melani and co-workers show that errors in inhalation technique are also common in specialized departments.[3] The trial investigated outpatient asthma and COPD patients in experienced centres.

An optimal inhalation technique is essential to the inhaled drug being successfully deposited in the lungs.[1] Most errors in inhalation techniques were shown in elderly patients, patients with a lower education level or lacking personnel instructions.

This leads to an increased risk for hospitalisation, admission to emergency department, therapy with corticosteroids, antibiotic therapy and poor disease control.[3]

Therefore, further trials clearly show that errors in inhalation technique can also occur in specialized departments and have to be optimized.

The German Airway League (Deutsche Atemwegsliga) has provided web-based, device-specific videos demonstrating proper inhalation techniques. Additionally, these videos aim to facilitate the teaching of correct inhalation and are universally accessible via the internet for patients and medical staff.

### Aim of the Trial

The trial of Malani and co-workers demonstrated that lacking personnel instruction from physicians or medical staff is associated with errors in inhalation technique.[3]

The aim of the current trial is to investigate the potential role of web-based videos [German airway league e.V., <http://www.atemwegsliga.de/richtig-inhalieren.html>] versus personell instructions by a qualified physician. It shall be demonstrated that a qualified personnel instruction and a web-based video is equal with regard to severe handling errors and may web-based videos are superior and can replace the personnel instruction.

**Patienten**

COPD patients who used either a pressurized metered-dose inhaler (pMDI), dry powder inhaler and/or soft mist inhaler will be included. Patients will be enrolled during a hospital stay at the Department of Pneumology, Cologne Merheim Hospital, University of Witten/Herdecke, Germany. Written informed consent will be obtained from all subjects.

Patients will be excluded if they meet any of the following exclusion criteria: acute respiratory failure as defined by a pH <7.35, breathing frequency >23/min at rest, need for supplemental oxygen of >3L/min. Neurologic, orthopaedic or cognitive conditions hindering inhalative treatment also serve as exclusion criteria and patients with an error index of less than 2 defined as a almost correct inhalation technique.

##### Study design

After Informed written consent was obtained from all subjects demographic data will be collected from all patients. Additionally, full bodyplethysmography will be performed to confirm diagnosis of COPD.

Individual patient-related errors will be assessed using standardized check-lists. Patients with 2 or more errors will be deemed to perform the inhalation process incorrectly without doubt. If patients use more than one device, individual handling errors will be assessed for each device.

All of these patients therefore received inhalation training. For this purpose, patients will be randomized amongst two groups, one in which they undergo a personal instruction in the full attendance of a physician, and the other in which they undergo web-based video teaching [German airway league e.V., <http://www.atemwegsliga.de/richtig-inhalieren.html>].

During the personal instruction, patients were shown the correct inhalation process using a demo device of the device with the individual highest error rate. In case of an equal error rate in two different devices patients will be allowed to choose one device for inhalation training. Patients will be next requested to practice all the steps of correct inhalation. Here, particular emphasis will be placed on the steps that the patients had originally performed incorrectly. Finally, patients get the opportunity to ask any outstanding questions.

After 24 hours, individual patient-related errors will be assessed again using the same standardized check-list (web-based video versus personnel instruction). In order to avoid investigator bias, both the initial and post-teaching error assessments will be performed by the same investigator

##### Checklists

In line with Melani et al. checklists for the assessment of correct inhalation were recently developed.[3] Therefore, we developed individual check lists for frequently used hand-held devices used for inhalation treatment. Ten items were formulated for each check list. Three major steps of the inhalation process were covered by each checklist: inhalation preparation, inhalation routine and closure of inhalation. The checklists were standardized to allow comparability amongst patients. Patients will be enrolled if they have more than 2 inhalation errors, defined as severe handling error.

##### Statistical analysis

The purpose of the study is to show that web-based video teaching is not inferior to personal instruction with respect to the probability of a severe handling error persisting, as defined by ≥2 errors described on the checklists. For the purpose of sample size calculation it has been assumed that following personal instruction, the probability of a severe handling error persisting would be 0.05. Non-inferiority is assigned if the probability that a severe handling error persisted after web-based video teaching was not higher than 0.15. Thus, the limit for non-inferiority is 0.1. At least 75 patients per group will be needed in order to show non-inferiority of the two methods on a one-sided alpha level of 0.025 with a power of 80%. Non-inferiority is statistically shown when the upper limit of the two-sided 95% confidence interval (CI) of the difference between error probabilities after subtracting video teaching from consent discussion is lower than 0.1. If the upper limit is lower than 0, superiority of the web based video teaching is shown.

Baseline characteristics will be descriptively compared between teaching methods. The results of the checklists will be descriptively presented for each device.

**Literatur**

1 Laube BL, Janssens HM, de Jongh FHC, et al. What the pulmonary specialist should know about the new inhalation therapies. Eur Respir J 2011; 37:1308-31.

2 Wieshammer S, Dreyhaupt J. Dry powder inhalers: which factors determine the frequency of handling errors? Respiration 2008; 75:18-25.

3 Melani AS, Bonavia M, Cilenti V, et al. Inhaler mishandling remains common in real life and is associated with reduced disease control. Respir Med 2011; 105:930-8.

4 Blackwelder WC. 'Proving the null hypothesis' in clinical trials. Control Clin Trials 1982; 3:345-53.
